# GL Beams Reinforced with Plywood in the Outer Layer

**DOI:** 10.3390/ma15113976

**Published:** 2022-06-02

**Authors:** Dorota Dziurka, Adam Derkowski, Marek Wieruszewski, Marcin Kuliński, Radosław Mirski

**Affiliations:** Department of Mechanical Wood Technology, Faculty of Forestry and Wood Technology, Poznań University of Life Sciences, Wojska Polskiego 28, 60-637 Poznań, Poland; adam.derkowski@up.poznan.pl (A.D.); marek.wieruszewski@up.poznan.pl (M.W.); marcin.kulinski@up.poznan.pl (M.K.); radoslaw.mirski@up.poznan.pl (R.M.)

**Keywords:** beams, glued elements, solid wood, plywood, mechanical properties

## Abstract

Glulam beams are increasingly used in the construction industry because of their high strength and the possibility of using round timber with smaller cross-sections. The load-bearing capacity of beams is strongly related to the quality of the outer layers and, in the case of wood, especially the tension zones. For these reasons, this study decided to replace the outer lamella with tensile plywood. The produced beams were subjected to static bending strength and modulus of elasticity evaluation. It was shown that the best static bending strength values were obtained for beams containing plywood in the tension layer. However, the change in structure in the tension zone of beams made of glued laminated timber results not only in an increase in the load capacity of elements produced in this way but also in a decrease in the range/range of the obtained results of bending strength. This way of modifying the construction of glued laminated beams allows a more rational use of available pine timber.

## 1. Introduction

The growth of the construction industry is causing this industry to look not only for alternative construction materials but also for the development of current ones. Engineering Wood Products (EWP) are construction-related materials. EWPs involve, among other things, obtaining a full-value product from a material that was initially unsuitable for specific applications due to its size or insufficient quality. Nowadays, there has been a development in Europe and worldwide in the manufacture and use of glulam, mainly GLT (Glued Laminated Timber). GLT has the typical characteristics of solid wood: low weight, good strength, resilience, durability, ease of processing, and the unique feature of being easy to shape cross-sections. Its cross-section has a layered structure, which allows the production of elements with variable section height, depending on the needs. The timber elements are glued with adhesives that guarantee high static and dynamic loads [1,2,3]. The high properties of GLT do not mean that the development of this product has been abandoned. It seems that one of the oldest ways of strengthening wood, or improving its performance characteristics, is to combine it with steel. Initially, steel was used to join individual elements, or as anchors to connect wood to other materials. However, quite a long time ago, there were already solutions where steel was used to reinforce timber in the sense that is now understood and accepted [4,5,6]. One of the first precursors of the use of steel as a wood reinforcing element was Dziuba at Poznań University [7], and the work is continued by Mirski [8]. Steel tendons can be used to strengthen newly designed beams and renovate existing ones [9,10,11]. An attractive solution, in which wood is instead a cladding for H-profile steel, was proposed by Duan and the team [12].

The idea of steel reinforcement is to introduce a stiffer material in the section, which increases the stiffness of the beam and thus reduces its deflection. Another way to increase the load-bearing capacity of glued laminated beams and timber is to use all kinds of carbon fiber strapping Glass Fiber Reinforced Polymer (GFRP). Composite systems are an excellent alternative to steel to increase the quality of beams made of wood. As a result of these actions, an increase in the modulus of elasticity is observed, and the increasing strength can even be doubled under very favorable conditions. The literature in this field is pervasive [13,14,15,16,17,18,19,20]. FPR or GFPR tapes do not result in an excessive increase in structural weight. Furthermore, in the case of the reconstruction of service echoes of already installed beams in the structure, this may be the only way.

In general, it does not matter which zone we reinforce for the sake of the result. In timber-concrete composite (TCC) systems, the timber is placed in the tension zone (which has much better resistance to bending and tension) and the concrete in the compression zone. Due to the low density of wood, the system obtains a better strength-density ratio than, for example, systems with steel. When concrete is reinforced with steel, the arrangement is often reversed. In this case, the concrete-steel composite is placed in the tension zone [21,22,23,24,25].

All the methods described above are related to the use of adhesives that allow strong bonding of the elements to be joined together. Most often, this adhesive is an epoxy resin. It is generally known that the quality of glued laminated beams, and glued and other wood materials produced, depends on the quality of the adhesive resin used [26,27,28]. However, the use of different adhesive agents can be technologically cumbersome. Moreover, especially when additional reinforcement is placed below the outer layer, its quality must also be relatively high. The use of wood materials, which generally have a uniform quality on the surface and cross-section, should allow easier production of glued laminated beams, and reduce the range of the obtained static bending strengths.

## 2. Materials and Methods

### 2.1. Manufacture of Beams

Beams were manufactured from pine timber approximately 40 mm thick, 138 mm wide, and 120 mm wide, and 40 mm and 22 mm thick. The length in both cases was 3500 mm. The timber was surface planed for better glue application before it was put into sets, ready for pressing. The beams were manufactured as asymmetric, in the sense that in the tension zone, the outer layer/lamella was either 21 mm thick plywood or 40 mm thick LVL. The beams were produced as eight layers, with a similar thickness of layers counting from the beam axis. As the plywood was 21 mm thick, the compression layer was pine timber, also 21 mm thick. The manufactured beams were marked so that when LVL face was used, the beam was marked LVL; when 138 mm wide pine timber and plywood were used, the manufactured beams were marked BB; and when 120 mm wide pine timber was used, the beams were marked BS.

Generally, pine sawn timber from the Olesno Forest District was used in the study. In the case of timber 138 mm wide, the modulus of elasticity was determined in a 4-point bending test. In contrast, the 120 mm wide timber was tested in a sawmill (Tartak Witkowscy, Wieluń, Poland), which determines the class C24 by the sonic measurement of the modulus of elasticity. The quality of the lamellas used and their distribution in the beam structure are presented in Table 1. The data presented herein show that the modulus of elasticity of timber 120 mm wide is slightly lower than that of timber 138 mm wide. The beams were cold press manufactured using MUF 1247 (Akzo Nobel, Amsterdam, Netherlands) adhesive at 220–240 g/m^2^ mixed with 2526 hardener (Akzo Nobel) at 10% of the resin dry weight. The mixture was prepared using the conditions in the laboratory hall. The glue was applied using a roller applicator. After loading the press, a pressure of 0.48 MPa was applied. The beams were in the press for at least 4 h.

### 2.2. LVL

**Figure 1:**
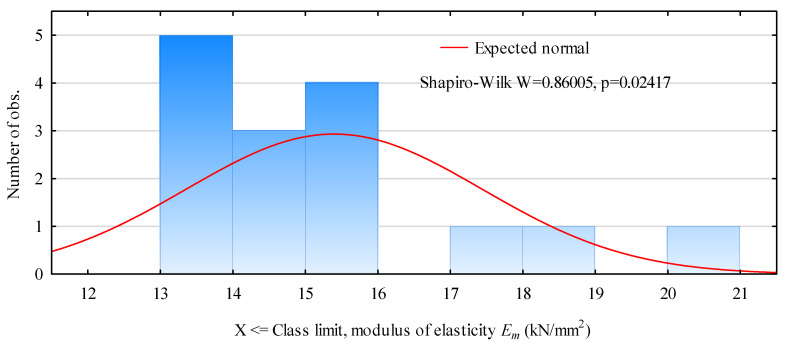
Histogram of the distribution of the modulus of elasticity of LVL belts used in the study.

LVL is a specialized type of plywood. LVL belts with a thickness of 40 mm and a width similar to that of pine timber were used. The moisture content of the LVL belts was similar to that of the timber and was approximately 11% ± 1%. The modulus of elasticity of LVL was also evaluated in a 4-point bending test, before the actual tests. The tension spacing was 980 mm and the arm length was 980 mm, so the system was tested at a spacing of more than 19 × belt height (the height of the tested belts was 151 mm). The evaluation was therefore conducted with sufficient spacing to determine the elastic modulus in the pure bending zone. The mean value of the elastic modulus was 13.89 kN/mm^2^ (SD = 1.97 kN/mm^2^). The results of the direct measurements are shown in Figure 1. They are generally concentrated in the range of 13–16 kN/mm^2^ and are, therefore, by the manufacturer’s declaration (Steico S.A., Czarna Woda, Poland). This level of modulus of elasticity should be considered high in comparison with the average modulus of elasticity of pine wood.

### 2.3. Plywood

**Figure 2:**
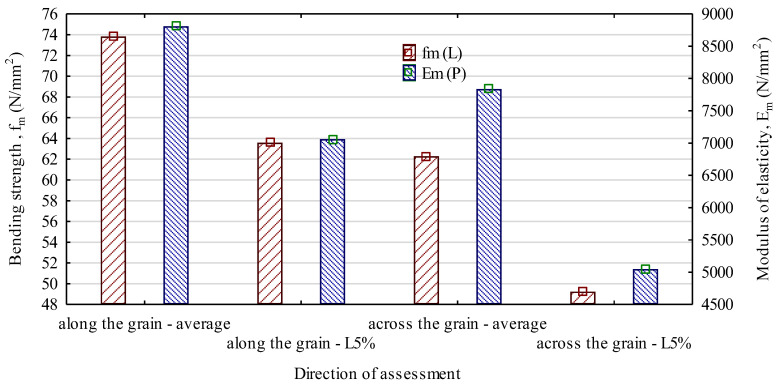
Properties of plywood used in the study: mean–average value for 15 samples, L5%—characteristic value (5-percentile value).

For the outer layer, we used plywood produced for the project by the company Biaform S.A. (Białystok, Poland). As the only one in Poland, this company can make plywood with a length of 3350 mm. Birch plywood was used in the tests. The thickness of waterproof plywood was 21 mm. Plywood bending strength (according to EN 310), determined based on 15 tests, is 74 N/mm^2^ for the lengthwise and 62 N/mm^2^ for the crosswise (Figure 2). These values are significantly higher than those determined for structural pine timber. The 5th percentile value shows a level similar to previously manufactured beams for the system across the fibers. The longitudinal modulus of elasticity, determined by the 3-point bending test, is 7.8–8.8 kN/m^2^; in the 4-point bending test, the average value of the modulus was 9.9 GPa, with a standard deviation of 0.64 GPa.

The moisture content of the plywood belts was slightly lower than the moisture content of the timber and was approximately 8% ± 1%. Since the length of the plywood belts was less than the length of the pine timber, the belts were extended. A piece of plywood approximately 15 cm long was attached to each end. The connection was made with the use of finger–jointing. The joint proved to be durable, as none of the joints were damaged.

### 2.4. Test

**Figure 3:**
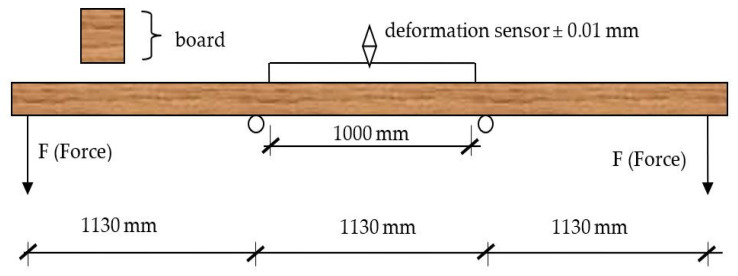
Scheme for flexural strength evaluation.

After a conditioning period of about two weeks, the beams were evaluated for flexural strength and modulus of elasticity in a four-pound bending test (Figure 3). Before assessing the mechanical properties, the moisture content of each beam was tested. Moisture content was determined using a HIT-3 resistance moisture meter.

The obtained direct measurements were statistically evaluated using Statistica version 13.0. The results were analyzed using the STATISTICA 13.0 package (Version 13.0, StatSoft Inc., Tulsa, OK, USA).

## 3. Results and Discussion

**Figure 4:**
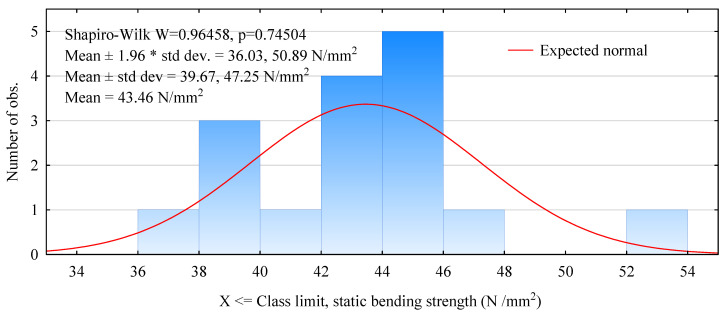
Histogram of static flexural strength of beams manufactured with LVL facing in the tension zone.

Static flexural strength is a fundamental quantity for evaluating the quality of wood materials. Figure 4 shows the distribution of the obtained static bending strengths of beams made with LVL facing. The results obtained are characterized by a normal distribution, with dominant strengths of 42–46 N/mm^2^. The strength distribution, except for one beam, is continuous. However, the results obtained are 36–48 N/mm^2^, resulting in an average strength of 43.4 N/mm^2^ (Figure 4). This is slightly lower than the previously observed value for non-wood tendon systems [8]. The declared strength of LVL is at a similar level, as the manufacturer reports 44–45 N/mm^2^. The strength of beams so manufactured is still more than 30% higher than beams manufactured without the modification of the face layer [29]. The 5-percentile value is 36.6 N/mm^2^. Thus, the quality of such a set is still as much as 85% higher than the basic set.

**Figure 5:**
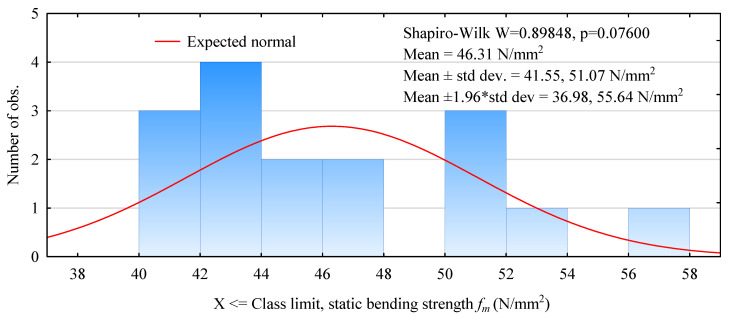
Histogram of static flexural strength of 138 mm beam width beams made with plywood face.

Figure 5 shows the distribution of the obtained static flexural strengths for beams made with 138 mm wide pine timber and a plywood face layer. This case, therefore, coincides with the example where LVL was used for the face. A very weak normal distribution characterizes the obtained static flexural strength results. In this case, strength values in 42–44 N/mm^2^ predominate. Two-thirds of the beams failed in the strength range of 40–48 N/mm^2^, and only five beams failed at the pressure responsible for strengths above 50 N/mm^2^. In contrast, the average strength of beams with plywood facing layers is as high as 46.3 N/mm^2^ (Figure 5), slightly higher than the LVL system. The 5th percentile value, on the other hand, is higher than that specified for the LVL beams and is 40.8 N/mm^2^.

**Figure 6:**
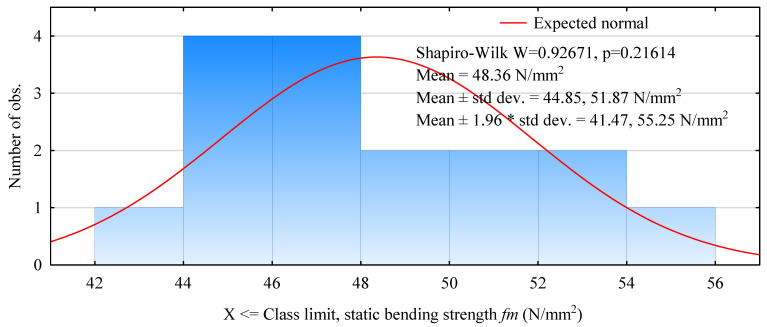
Histogram of static flexural strength of 120 mm beam width beams made with plywood face.

Reducing the width of the beams and using a different sorting technique did not significantly affect the quality of the observed relationships. In this case, the strengths in the range 44–48 N/mm^2^ predominate. As many as half of the tested beams failed in this strength range. The strength distribution is normal and is in the range of 42–56 N/mm^2^ (Figure 6). The average static flexural strength for this beam type is as high as 48.4 N/mm^2^. The basis for both types of beams is pine timber from the same manufacturer and graded similarly. Additionally, the 5th percentile value of 43.6 N/mm^2^ is higher than previously observed.

**Figure 7:**
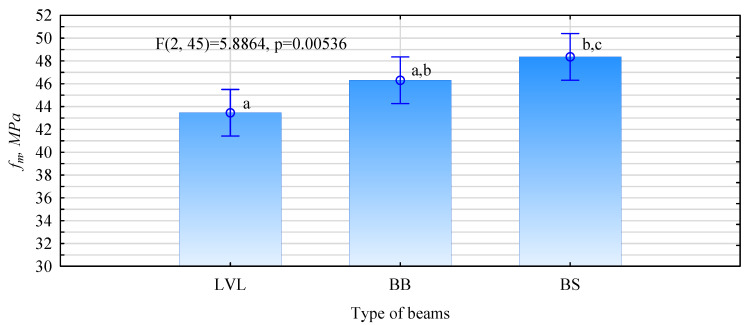
ANOVA analysis of static flexural strength of beams with reinforced tensile zone face.

Statistical ANOVA analysis of static bending strength indicates that the manufactured beams differ significantly in the values obtained for this mechanical property (Figure 7). It should be noted that there are no statistical differences in the strength of beams produced from 138 mm and 120 mm wide timber with plywood belts, even though the 120 mm wide timber was classified according to the dynamic elastic modulus and the 138 mm wide timber according to the mechanical modulus. In contrast, there are no statistical differences in the layout of the 138 mm timber. The only significant difference is between the LVL beams and the thinner plywood-faced beams. However, it is difficult to determine the cause of this phenomenon.

**Figure 8:**
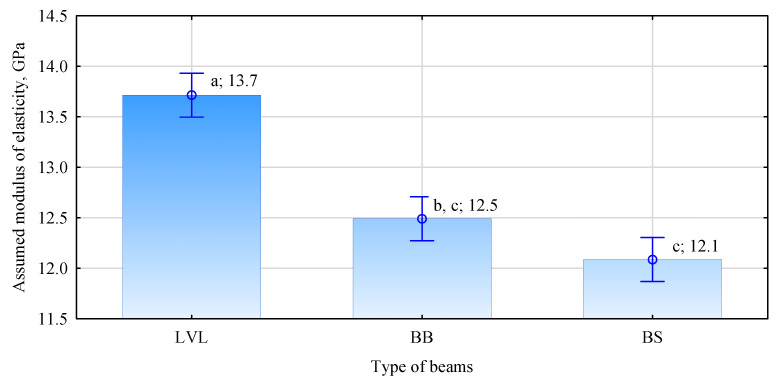
Estimated modulus of elasticity of beams with a reinforced face of the tension zone.

Analyzing the preliminary assumptions about the load-carrying capacity of manufactured beams, it turns out that beams should characterize the highest parameters with LVL in the outer layer and the lowest by beams with 120 mm wide plywood (Figure 8). The assumed quality of the fabricated beams is based on a rough analysis based on the formula given by Bodig and Jayne. Earlier work describes these assumptions in detail [29]. However, it should be mentioned here that it is based on the determination of the equivalent (effective) modulus of elasticity for a multilayer system based on knowledge of the modulus of elasticity of individual layers. Another assumption is the high correlation between the modulus of elasticity of wood, including glued laminated timber, and static bending strength; although this is assumed by the interested community, the authors have doubts about it.

**Figure 9:**
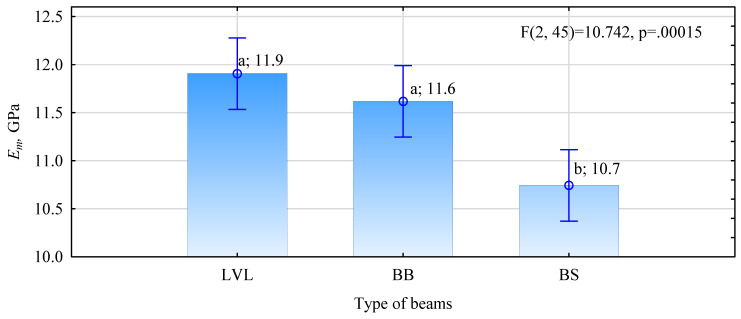
Tested modulus of elasticity of beams with a reinforced face of the tension zone.

On the other hand, the data presented in Figure 7 and Figure 8 or Figure 9 show that the opposite effect was obtained. However, it should be remembered that the tests were performed on a small sample. Only 16 beams of each type were evaluated. Perhaps less significantly, the specified modulus of elasticity is on average about 11% less than that estimated from the knowledge of the modulus of elasticity of each piece. In contrast, no significant effect of determining the modulus of elasticity of the timber on the modulus of elasticity of the fabricated beams was observed. The changes are similar in both analyzed cases.

The available literature is inconclusive about the quality of the solution used but is consistent with the observations from the presented studies. Hayashi and Miyatake [30] studied beams made of sugi (Cryptomeria japonica D. Don), in which the layer/tensile zone was made of LVL. They conclude that an increase in stiffness of the beams so produced was observed, but no increase in bending strength was observed. Enders-Comberg’s work [31], which investigated similar beams (GL + Beech LVL), showed an approximately 50% increase in strength, and a troublesome-to-estimate increase in stiffness (between 10% and 70%). Thus, already, based on these two works, it can be seen that the quality of the produced beams depends, not only on the quality in the discussed cases LVL, but also on the quality of the timber used. This is also confirmed by Kržan’s study [32], which shows that when the quality of the outer layer (in this case, beech) was high, the internal spruce lamellas were responsible for the destruction of the beam. Even very high LVL properties can therefore be masked by low-quality wood. Other reports show that the use of plywood [33] or LVL [34,35,36] significantly affects the quality of manufactured elements, but the search for layouts in which the available raw material is used more advantageously is an ongoing issue.

## 4. Conclusions

The advantage in the production of glued elements compared with solid wood is that we can more easily obtain both the cross-sectional dimensions and the length dimension, much larger than the linear dimensions of the wood from which the product is obtained. On the other hand, joining wood with wood-based materials also seems to be beneficial because the entire gluing process is prepared. The following observations were formulated based on the conducted research:

– The method of sorting timber does not significantly affect the quality of beams reinforced in the plywood tension zone;
– The value of the modulus of elasticity of beams glued in layers turned out to be about 10% lower than expected based on the evaluation of the modulus of elasticity of individual lamellas/pieces of timber and plywood;
– Significantly better static flexural strength values were obtained for beams containing plywood in the tension layer, which may be due to the species of the wood used (birch plywood, pine LVL);
– The change in structure in the tension zone of beams made of glued laminated timber results not only in an increase in the load capacity of elements produced in this way but also in a decrease in the range/range of the obtained results of bending strength;
– Based on the quality of the sawn timber, it was estimated that the characteristic static bending strength of the produced beams would be in the range of 26–32 MPa. However, the obtained values are 36.6–43.6 MPa; therefore, they are in the safe range.

## Figures and Tables

**Table 1:** Quality of pine timber used.

Type	Width mm	Lam. 2	Lam. 3	Lam. 4	Lam. 5	Lam. 6	Lam. 7	Lam. 8
		Static Modulus of Elasticity (GPa)						
LVL	138	13.6	13.3	12.7	12.7	13.3	13.6	13.9
BB	138	13.7	12.9	12.4	11.8	12.4	12.9	13.6
BS	120	Dynamic Modulus of Elasticity (GPa)						
		12.92	11.04	9.14	9.21	11.02	12.90	13.00

## Data Availability

The data presented in this study are available on request from the corresponding author.
